# Nitrate Respiration Protects Hypoxic *Mycobacterium tuberculosis* Against Acid- and Reactive Nitrogen Species Stresses

**DOI:** 10.1371/journal.pone.0013356

**Published:** 2010-10-26

**Authors:** Mai Ping Tan, Patricia Sequeira, Wen Wei Lin, Wai Yee Phong, Penelope Cliff, Seow Hwee Ng, Boon Heng Lee, Luis Camacho, Dirk Schnappinger, Sabine Ehrt, Thomas Dick, Kevin Pethe, Sylvie Alonso

**Affiliations:** 1 Novartis Institute for Tropical Diseases, Singapore, Singapore; 2 Immunology Programme, Department of Microbiology, National University of Singapore, Singapore, Singapore; 3 Department of Microbiology and Immunology, Weill Cornell Medical College, New York, New York, United States of America; Institut de Pharmacologie et de Biologie Structurale, France

## Abstract

There are strong evidences that *Mycobacterium tuberculosis* survives in a non-replicating state in the absence of oxygen in closed lesions and granuloma *in vivo*. In addition, *M. tuberculosis* is acid-resistant, allowing mycobacteria to survive in acidic, inflamed lesions. The ability of *M. tuberculosis* to resist to acid was recently shown to contribute to the bacillus virulence although the mechanisms involved have yet to be deciphered. In this study, we report that *M. tuberculosis* resistance to acid is oxygen-dependent; whereas aerobic mycobacteria were resistant to a mild acid challenge (pH 5.5) as previously reported, we found microaerophilic and hypoxic mycobacteria to be more sensitive to acid. In hypoxic conditions, mild-acidity promoted the dissipation of the protonmotive force, rapid ATP depletion and cell death. Exogenous nitrate, the most effective alternate terminal electron acceptor after molecular oxygen, protected hypoxic mycobacteria from acid stress. Nitrate-mediated resistance to acidity was not observed for a respiratory nitrate reductase NarGH knock-out mutant strain. Furthermore, we found that nitrate respiration was equally important in protecting hypoxic non-replicating mycobacteria from radical nitrogen species toxicity. Overall, these data shed light on a new role for nitrate respiration in protecting *M. tuberculosis* from acidity and reactive nitrogen species, two environmental stresses likely encountered by the pathogen during the course of infection.

## Introduction

Two billion individuals worldwide are currently infected with *Mycobacterium tuberculosis*, the etiological agent of human tuberculosis (TB) [1]. Among these, only 10% suffer from active TB, implying that the majority of *M. tuberculosis* infections result in latent disease which may be reactivated under certain circumstances, including co-infection with HIV and other immuno-suppressive conditions [2]. Latent TB has been defined as an asymptomatic phase of the disease during which mycobacteria remain dormant. However, recent evidences indicate that rather than a binary distribution between active and latent disease, tuberculosis should be seen as a wide continuous spectrum of infection outcomes characterized by a range of lesions that provide different microenvironments with different abilities to support bacterial replication, persistence or killing [3].

The location of latent bacilli remains to be formally demonstrated; current paradigm is that quiescent bacilli reside within fibrotic granulomatous lesions in the lung where *M. tuberculosis* has become dormant in response to hypoxic conditions [4]–[6]. The physiology of hypoxic non-replicating *M. tuberculosis* has been studied *in vitro* in the Wayne model of persistence in which mycobacterial cultures are subjected to self-generated oxygen depletion in sealed containers [7]. In this model, mycobacteria undergo drastic changes in their energetic and metabolic status [8], [9]. However, the molecular mechanisms involved in the survival of non-replicating hypoxic mycobacteria remain largely unknown. The DosR/DosT two-component system has been shown to be essential for the adaptation of mycobacterial cells to survive under anaerobic conditions [10], [11], although the role of the DosR regulon in the adaptive response of *M. tuberculosis* to hypoxia has recently been reassessed [12]. In addition to hypoxia, *M. tuberculosis* is exposed and responds to many other environmental stresses including nutrient deprivation, iron restriction, mild acidity, and reactive nitrogen and oxygen species [3]. Studies have shown that mycobacterial transcriptional responses to these environmental cues often involve overlapping gene sets [13]–[16], calling for cautious interpretation of data when analyzing the role of a specific exogenous stimulus in the disease progression.

Acidity is believed to be an important environmental parameter encountered by *M. tuberculosis* during its host infection, in particular in inflamed lesions where necrotic activated macrophages release substantial amounts of their acidic phagolysosomal content. This view is supported by the high *in vivo* killing activity of pyrazinamide, an antitubercular drug only active at acidic pH [17]. *M. tuberculosis* is capable of responding transcriptionally to acidic pH *in vitro* [16] and has the means to resist to acidity, although the molecular mechanisms have yet to be elucidated [18]. A recent work has identified a membrane-bound protein directly involved in *M. tuberculosis* intrabacterial pH maintenance, and established for the first time a link between acid resistance and virulence [19].

In this work, the mechanisms contributing to acid-resistance were investigated. We show that the ability of *M. tuberculosis* to resist acid stress is oxygen-dependent. In the absence of an efficient terminal electron acceptor, mild acidic conditions promoted the dissipation of the protonmotive force, rapid ATP depletion and cell death. Survival under hypoxic acidic conditions was restored in the presence of nitrate that is acting as an effective terminal electron acceptor (TEA) for anaerobic respiration. In addition, we show that nitrite, a toxic by-product of nitrate respiration, was largely secreted in the extracellular milieu rather than being detoxified intracellularly by the bacillus. Lastly, nitrate respiration was found equally important in protecting *M. tuberculosis* against reactive nitrogen species which are likely to be encountered by the pathogen in the infected host.

## Materials and Methods

### Bacterial cells, culture conditions and growth media

*M. tuberculosis* H37Rv (ATCC # 27294), derived-mutant and complemented strains were maintained in Dubos complete medium which consists of Dubos broth (Difco) supplemented with 0.05% (v/v) Tween-80, and 10% Dubos Medium Albumin (Difco). The pH of the medium was adjusted by addition of pre-determined volumes of 1.25 M tri-sodium citrate buffer and 10 M hydrochloric acid. Where indicated, the culture medium was supplemented with exogenous nitrate or nitrite by addition of sodium nitrate or sodium nitrite solutions, respectively. When appropriate, hygromycin and kanamycin were added at 80 ug/ml and 20 ug/ml, respectively.

Enumeration of bacteria was performed by plating on Middlebrook 7H11 Agar (Difco) containing 0.5% (v/v) glycerol and the number of colony-forming units (CFU) was determined after 16 days incubation at 37°C.

### Anaerobic shift-down assay

Aerated pre-cultures in Dubos complete medium were harvested at mid-log phase, washed twice in phosphate-buffered saline (PBS) supplemented with 0.05% Tween-80 (PBS-T), and resuspended in Dubos complete medium at a final OD_600nm_ of 0.1. The bacterial suspension was then distributed in 24-well tissue culture plates (1 ml/well). Methylene blue (1.5 ug/ml) was added as an indicator of oxygen depletion in control wells. The plates were incubated in air-tight anaerobic jars (BioMerieux) with Anaerogen and Campygen gas packs (Oxoid), to generate anaerobic and microaerophilic atmospheric conditions, respectively. Atmospheric oxygen depletion was indicated by the anaerobic indicator strip (BD Diagnostics). Hypoxia was typically achieved within 24 hours after incubation under anaerobic conditions, as witnessed by the complete decolorization of the oxygen sensor methylene blue. Survival was monitored by CFU count up to 10 days after methylene decolorization. Each experimental sample consisted of triplicate wells.

### Generation of *ΔnarGH* and *ΔnirBD M. tuberculosis* mutants

The Δ*narGH* and Δ*nirBD* mutants were generated in the H37Rv background by homologous recombination using suicide plasmid backbone pYUB854 and as described previously [20]. Briefly, regions flanking the target genes were amplified by PCR and cloned into pYUB854 containing the Pa*cI*-digested *lacZ/sacB* insert from pGOAL17 [21]. *M. tuberculosis* H37Rv bacteria were electroporated with 1 ug of the UV-irradiated plasmid solutions as described previously [9]. Hygromycin resistant white colonies were selected and correct deletion was verified by PCR and southern blot analysis. Complementation of the Δ*narGH* mutant was performed by introducing the *narGHJI* operon cloned into the integrative plasmid pMV306 [22].

### ATP, nitrite production and membrane potential

Mycobacterial suspensions were centrifuged at 4°C and the bacterial pellets were resuspended in 200 ul Dubos complete medium. Intracellular ATP production was measured using the BactTiter-Glo Microbial Cell Viability Assay kit (Promega) as described before [9]. Intracellular and extracellular nitrite production was quantified using the Griess reagent kit (Molecular Probes) following the manufacturer's instructions. Briefly, 130 ul water, 150 ul sample (cell lysate or supernatant) and 20 ul Griess reagent were combined in each well of a 96-well tissue culture plate and the reaction was incubated for 30 min before absorbance reading at 548 nm. Absorbance readings were extrapolated to absolute nitrite concentrations by reference to a nitrite standard curve (ranging from 12.5 to 100 uM). Membrane potential of *M. tuberculosis* cells was measured using the BacLight Bacterial Membrane Potential Kit (Molecular Probes) as described previously [9]. Briefly, cells were harvested, washed with PBS-T, and incubated with 3, 3′-diethyloxacarbocyanine iodide (DiOC_2_) for 30 min. The membrane potential of positive control samples was collapsed by the addition of the ionophore carbonyl cyanide 3-chlorophenylhydrazone (CCCP) for 30 min prior to the addition of DiOC_2_. The green and red mean fluorescence intensities (MFI) were measured by flow cytometry using FACS Calibur (BD Biosciences) and data were analyzed using CellQuest Pro (BD Biosciences). The cell membrane potential was defined as the ratio of red to green MFI values.

### Statistics

Statistical significance was assessed by the Student's *t*-test, and two-tailed *p* values of less than 0.05 were considered statistically significant.

## Results

### *M. tuberculosis* acid resistance is oxygen-dependant

*M. tuberculosis* resistance to acidity was studied under aerobic, microaerophilic and hypoxic conditions. *M. tuberculosis* H37Rv was inoculated into 24-well plates containing culture medium buffered at neutral (6.6) or mild acidic (5.5) pH and exposed to aerobic, microaerophilic or hypoxic atmosphere, the latter being typically achieved within 24 hours.

**Figure 1:**
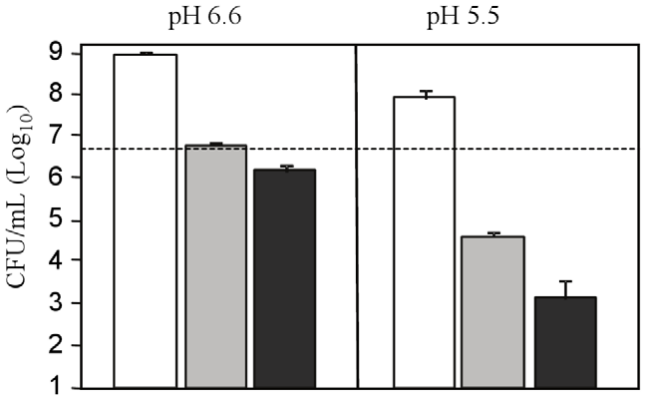
*M. tuberculosis* viability under hypoxic acidic conditions. *M. tuberculosis* H37Rv bacteria were incubated under aerobic (open bar), microaerophilic (grey bar) or anaerobic (black bar) conditions in Dubos medium adjusted to pH 6.6 or 5.5 as indicated. After 10 days incubation, bacterial cultures were plated for colony counting. The dotted line represents the average number of bacteria present in the inoculum at day 0. Results are expressed as the means of Log_10_ CFU/ml ± SD of triplicates.

At neutral pH, hypoxic and microaerophilic mycobacteria did not multiply but survived without significant viability loss for up to 10 days (Fig. 1). Instead and as expected, the bacterial cells multiplied more than 10-fold at neutral pH in the presence of oxygen. In mild acidity (pH 5.5), mycobacteria still multiplied efficiently in the presence of oxygen, illustrating *M. tuberculosis* acid resistance property previously described [18], [19] (Fig. 1). However, under anaerobic conditions, *M. tuberculosis* was found to be exquisitely sensitive to acid with only 1% of the original inoculum recovered at 10 days post-inoculation at pH 5.5 (Fig. 1). Interestingly, a similar phenotype was observed under microaerophilic conditions. These observations thus indicated that *M. tuberculosi*s ability to cope with acidity is oxygen-dependant, linking the respiratory functions to acid resistance.

### Collapse of the membrane potential precedes cell death in hypoxic acidic *M. tuberculosis*

**Figure 2:**
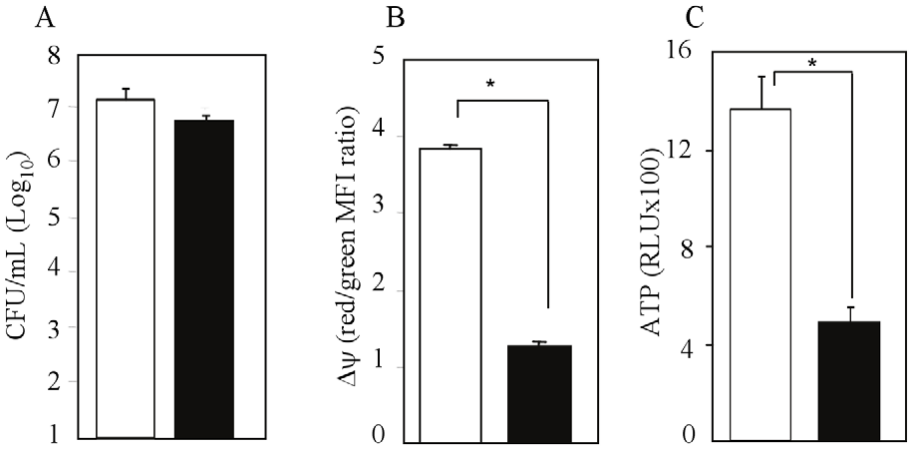
Membrane potential and ATP production in *M. tuberculosis* grown in hypoxic and mild acidic conditions. *M. tuberculosis* H37Rv bacteria were incubated under hypoxia and at pH 6.6 (open bar) or pH 5.5 (black bar). After 4 days incubation at 37°C, the bacterial suspensions were plated for colony counting (A), or were processed for (B) measurement of Δψ as an indicator of the membrane potential, or (C) determination of intracellular ATP. Results are expressed as the means ± SD of triplicates. MFI, Mean fluorescence intensity; RLU, relative luminescence units. *, *p*<0.005.

To further characterize the link between respiration and acid resistance in *M. tuberculosis*, the energetic status of the bacterial cells was determined at neutral and mild acidic pH under hypoxic conditions. Membrane potential and intracellular ATP level were measured as early as 4 days after the anaerobic shift-down, where *M. tuberculosis* viability at both pH is still comparable (Fig. 2A). The membrane potential of hypoxic mycobacteria incubated at pH 5.5 was largely dissipated when compared to cells incubated at pH 6.6 (Fig. 2B). In addition, the intracellular pool of ATP was reduced by more than 80% (Fig. 2C). These data indicated that in absence of oxygen and at mild acidic pH, the membrane potential of *M. tuberculosis* is compromised, thereby resulting in disruption of the protonmotive force and in reduced ATP production through respiration.

### The availability of an efficient exogenous alternate terminal electron acceptor protects hypoxic *M. tuberculosis* from acid-mediated killing

**Figure 3:**
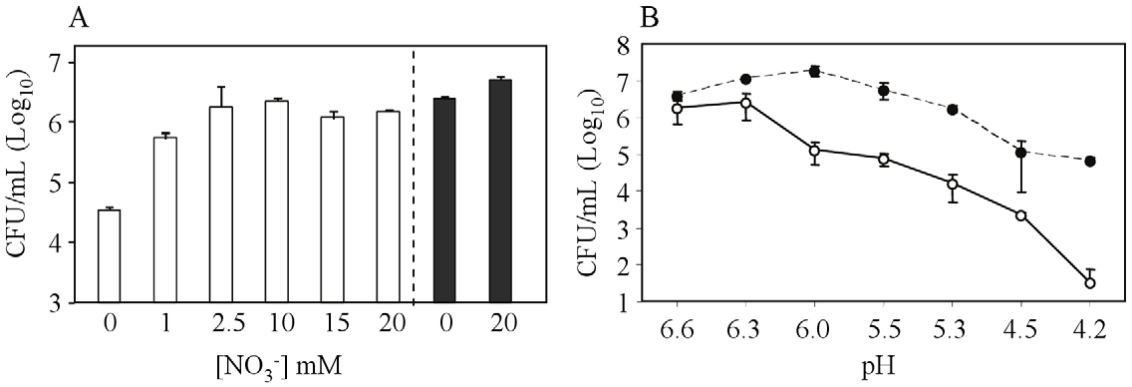
Role of exogenous nitrate in *M. tuberculosis* viability under hypoxic and mild acidic conditions. (A) *M. tuberculosis* viability at pH 5.5 (open bar) or pH 6.6 (black bar) in the presence of increasing concentrations of exogenous nitrates. (B) *M. tuberculosis* viability in the presence (black cirle) or absence (open circle) of 20 mM of exogenous nitrate and at increasingly acidic pH. After 10 days incubation, the bacterial suspensions were plated for colony counting. Results are expressed as the means of Log_10_ CFU/ml ± SD of triplicates.

In absence of oxygen, bacteria use alternative terminal electron acceptors (TEAs) whose nature varies depending on the composition of the culture medium and on the microorganism species. With a relatively high redox potential, nitrate represents the most efficient TEA after oxygen for a variety of facultative anaerobes, including *M. tuberculosis* [23]. We thus investigated whether through anaerobic respiration, exogenous addition of nitrate helps protect hypoxic *M. tuberculosis* from an acid challenge. Increasing concentrations of nitrate were added to the culture medium, and *M. tuberculosis* viability was determined at 10 days post-inoculation. At pH 5.5 and under hypoxia, exogenous addition of 2.5 mM NO_3_ and above resulted in cell viability comparable to that observed at neutral pH (Fig. 3A). Moreover, addition of exogenous nitrate significantly enhanced cell viability across a range of increasing acidic pH conditions (Fig. 3B).

**Figure 4:**
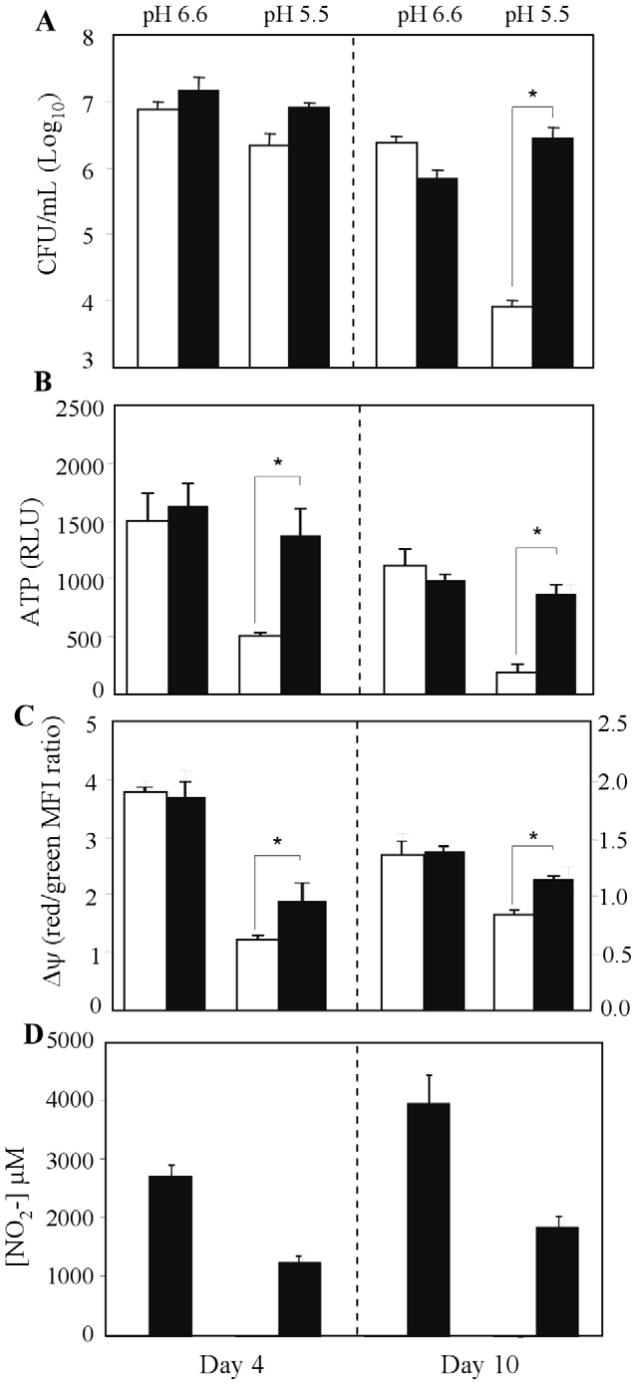
ATP and nitrite production in the presence of exogenous nitrate. *M. tuberculosis* viability (A), ATP production (B), membrane potential (C) and nitrite production (D) were assessed under hypoxic conditions at neutral (6.6) or mild acidic (5.5) pH, in the presence (black bar) or absence (open bar) of 20 mM nitrate after 4 and 10 days incubation period. Results are expressed as the means ± SD of triplicates. RLU, relative luminescence units; MFI, Mean fluorescence intensity. *, p<0.005.

The levels of intracellular ATP were measured in mycobacteria grown in the presence or absence of exogenous nitrate. At pH 6.6, the level of intracellular ATP was found nitrate-independent (Fig. 4A&B). In contrast, the level of intracellular ATP measured at pH 5.5 in the presence of nitrate was comparable to that observed at neutral pH (Fig. 4A&B), correlating with the enhanced cell viability observed. Consistently, the membrane potential was significantly improved in the presence of exogenous nitrate at pH 5.5, although not fully restored when compared to the membrane potential measured at neutral pH (Fig. 4C). These data thus suggested that partial restoration of the membrane potential by exogenous nitrate at pH 5.5 is sufficient to lead to the production of ATP levels and cell viability comparable to that measured at neutral pH.

Altogether, these data demonstrated that the presence of exogenous nitrate protected hypoxic mycobacteria from acid-mediated killing, and correlated with the maintenance of high level of intracellular ATP and partial restoration of the membrane potential, strongly suggesting a role for nitrate in *M. tuberculosis* acid resistance through anaerobic respiration.

Importantly, increased concentrations of nitrite (NO_2_), a by-product of nitrate respiration, were detected in the nitrate-supplemented culture media (Fig. 4C), further supporting the hypothesis that nitrate acts as TEA for *M. tuberculosis* anaerobic respiration in acidic condition.

### The respiratory nitrate reductase is required for acid resistance of hypoxic nonreplicating *M. tuberculosis*

**Figure 5:**
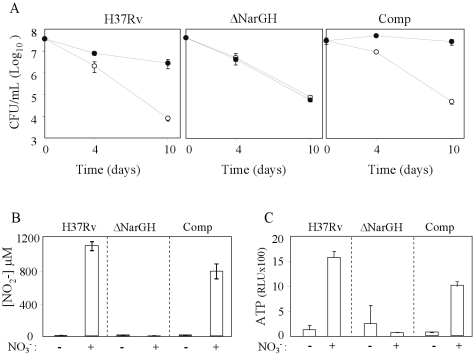
Viability, nitrite and ATP production of a *narGH* KO mutant under hypoxic acidic conditions. (A) Viability profiles of the wild-type (WT), *Δ*NarGH mutant and complemented (Comp) *M. tuberculosis* strains in the absence (open circle) or presence (dark circle) of 20 mM nitrate. (B) Nitrite and (C) intracellular ATP production measured for WT, ΔNarGH and Comp strains at day 10 in the absence (−) or presence (+) of 20 mM nitrate (NO_3_^−^). Results are expressed as the means ± SD of triplicates. RLU, relative luminescence units.

*M. tuberculosis* expresses a respiratory membrane bound-nitrate reductase [23], [24]. A *M. tuberculosis* mutant deleted for the *narGHJI* locus and its complemented counterpart were thus constructed. The mutant, complemented and parental strains all displayed comparable sensitivity to acid stress under hypoxic conditions (Fig. 5A). However, the addition of exogenous nitrate failed to protect the *Δ*NarGH mutant from acid-mediated killing, demonstrating that the respiratory nitrate reductase is required (Fig. 5A). Consistently, no increase in intracellular ATP concentration and no nitrite production were detected in the culture medium of the *Δ*NarGH mutant (Fig. 5B). In contrast, high CFU counts and ATP levels (Fig. 5A), as well as production of nitrite in the culture medium (Fig. 5B) were measured for the parental and complemented strains in the presence of exogenous nitrate. Altogether, these data demonstrated that under hypoxic acidic conditions, reduction of nitrate into nitrite by the respiratory nitrate reductase NarGHJI confers acid resistance to hypoxic *M. tuberculosis* through anaerobic respiration.

### Protection against nitrite toxicity is mediated through active export of nitrite outside the bacterial cell

**Figure 6:**
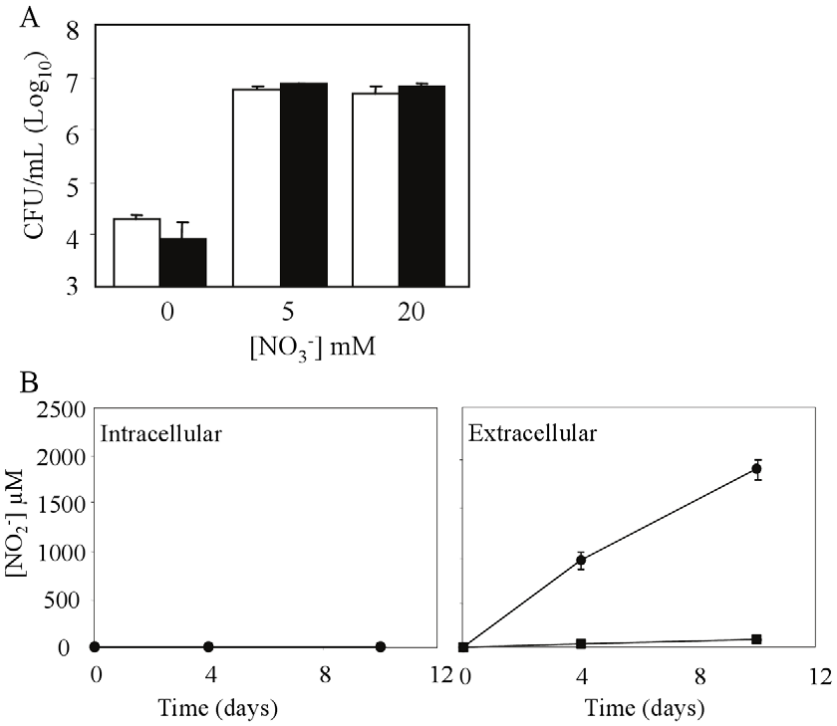
Role of NirBD in nitrite detoxification. (A) Viability of wild-type (open bar) and ΔNirBD (black bar) *M. tuberculosis* strains under hypoxia and at pH 5.5, in the presence of increasing concentrations of nitrate. Bacterial suspensions were plated after 10 days incubation for colony counting. (B) Intracellular (left panel) or extracellular (right panel) concentrations of nitrite after 10 days incubation of wild-type H37Rv bacteria under hypoxic mild acidic (pH 5.5) conditions, and in the absence (black square) or presence (black circle) of 20 mM nitrate in the culture medium. Results are expressed as the means ± SD of triplicates.

Nitrite is a toxic by-product of nitrate respiration. In enterobacteria, NirBD is required to detoxify nitrite produced intracellularly during nitrate respiration [25]. The *M. tuberculosis* NirBD homologue has been proposed to catalyze the reduction of nitrite to ammonium [26], [27], and we hypothesized that such enzymatic activity may help prevent the intracellular accumulation of toxic levels of nitrite arising from nitrate respiration under hypoxia and at acidic pH. A *M. tuberculosis* mutant deleted for the *nirBD* locus was thus constructed (ΔNirBD mutant), and its resistance to acid was tested under hypoxic conditions in the presence or absence of exogenous nitrate. Both the wild type and *Δ*NirBD mutant strains displayed comparable viabilities (Fig. 6A), indicating that the NirBD complex is not required for nitrate-dependent protection from acid-induced death under hypoxia. It also suggests that nitrite produced upon nitrate reduction through the NarGH complex is not further reduced into ammonium by the NirBD complex. Accumulation of intracellular nitrite being potentially toxic for the bacterial cell, we therefore hypothesized that nitrite may thus be excreted. To test this possibility, the amount of extracellular and intracellular nitrite was quantified. More than 90% of total nitrite produced was found in the extracellular milieu (Fig. 6B), suggesting that in *M. tuberculosis*, export of endogenous nitrite produced upon nitrate respiration likely constitutes the main strategy to protect the bacterial cell from nitrite toxicity.

### Nitrate respiration protects hypoxic *M. tuberculosis* from radical nitrogen species toxicity

Since nitrite does not seem to be significantly converted into ammonium but rather excreted outside the bacterial cell, its rapid conversion to nitric oxide (NO) at acidic pH [28] might still be harmful to *M. tuberculosis*. We thus tested the resistance of hypoxic *M. tuberculosis* to a NO stress at acidic pH. In this assay, nitrite, which at acidic pH is stochiometrically converted into NO, was used as a source of NO, as described elsewhere [28].

**Figure 7:**
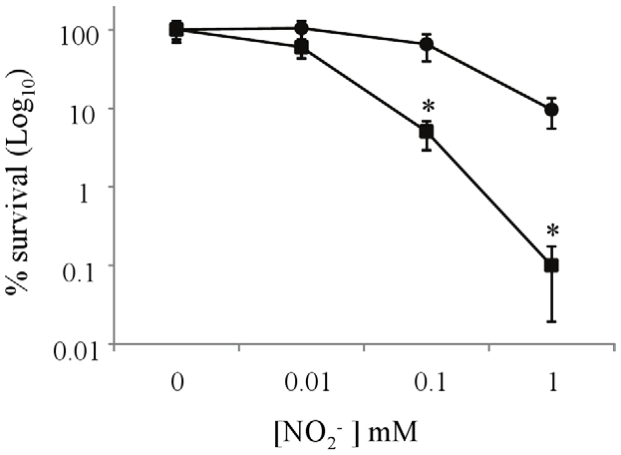
Nitrite toxicity under hypoxic and acidic conditions. *M. tuberculosis* viability in the presence of increasing concentrations of exogenous nitrite under hypoxia and at pH 5.5 in the absence (black square) or presence (black circle) of 2.5 mM nitrate. Bacterial suspensions were plated after 10 days incubation for colony counting. Results are expressed as the average of triplicates ± SD in percentage (%) of survival (in Log_10_ CFU/ml) compared to the survival obtained in absence of nitrite. *, p<0.005.

Under hypoxia and at neutral pH, addition of increasing concentrations of nitrite did not affect significantly *M. tuberculosis* viability (data not shown), consistent with the fact that at neutral pH, nitrite is not efficiently converted into NO and is therefore not toxic for the bacterial cell. In contrast, at pH 5.5, 0.1 mM nitrite and above led to a significant drop in *M. tuberculosis* viability with more than 95% cell death (Fig. 7). Interestingly, the presence of exogenous nitrate prevented such viability loss (Fig. 7), implicating that nitrate respiration protects hypoxic *M. tuberculosis* from NO stress in hypoxic nonreplicating *M. tuberculosis*.

## Discussion

Latent TB has been characterized by the presence of nonreplicating tubercle bacilli which are resistant to most of the anti-tubercular drugs currently used to treat the active disease [3], [9]. A better understanding of how *M. tuberculosis* is able to persist in its host, presumably in a hostile environment, for extended periods of time, will thus certainly facilitate the development of effective therapeutic strategies to eradicate latent tuberculosis. Hypoxia and acidity are two major environmental parameters encountered by the bacilli during the course of infection within granulomatous, inflamed lesions, as well as in the phagolysosomes of activated macrophages.

Whereas numerous studies have focused on *M. tuberculosis* response to hypoxia, fewer reports are available on the capability of *M. tuberculosis* to resist to acid. The isolation of acid-sensitive mutants suggested that *M. tuberculosis* has the means to resist to acid [18], [19], [29]. Such mutants displayed a defect in various cell wall functions and biosynthesis, Mg^2+^ transporter, pore-forming protein, and other membrane-bound proteins suggesting that the cell envelope clearly plays an important role in *M. tuberculosis* resistance to acid and maintenance of the intrabacterial pH. However, the actual molecular mechanisms involved in *M. tuberculosis* acid resistance have yet to be deciphered. Interestingly, the well-characterized mechanisms responsible for acid resistance in Gram negative bacteria have yet to be described in *M. tuberculosis*, including acid tolerance response (ATR), potassium-proton antiporters, amino acid decarboxylases and F_0_F_1_ ATPases [18].

Here, we provide evidence that *M. tuberculosis* resistance to acid involves the respiratory activity. We showed that *M. tuberculosis* resistance to acid is oxygen-dependent. Killing under hypoxia and mild acidity (pH 5.5) correlated with depolarization of the cell membrane and a drop of the intracellular ATP level. However, *M. tuberculosis* survival was greatly enhanced in the presence of exogenous nitrate, and paralleled with the maintenance of cell membrane potential and high levels of intracellular ATP. Furthermore, a mutant impaired in its respiratory NarGH nitrate reductase activity was not rescued upon addition of exogenous nitrate. Altogether, these data demonstrated that acid resistance in *M. tuberculosis* is dependent on the respiratory activity, hence on the protonmotive force, and that in absence of oxygen, nitrate acts as an effective terminal electron acceptor (TEA) to protect hypoxic mycobacteria from acid challenge.

In a previous work, we suggested that endogenous fumarate may be used as a TEA in hypoxic nonreplicating mycobacteria at neutral pH [9]. However, its low redox potential makes fumarate a much less efficient TEA compared to oxygen; it is thus likely that fumarate is not capable of maintaining effectively *M. tuberculosis* membrane potential and generating sufficiently high ATP levels under acidic conditions. These observations thus point to a role of nitrate respiration in protecting hypoxic mycobacteria specifically during acidic conditions.

The nitrate reductase system has been linked to *M. tuberculosis* virulence before; high nitrate reductase activity has been correlated with increased virulence of some *M. tuberculosis* lineages and their evolutionary success [30]. Also, the *narGH* locus was found actively transcribed in granulomas from the lungs of TB patients [31]. However, we (this study, data not shown) and others [32] did not find that a *nar*GH KO *M. tuberculosis* mutant was significantly attenuated upon nasal infection of immunocompetent mice. This absence of phenotype is likely accounted for by the fact that granulomatous lesions and cavities formed in *M. tuberculosis*-infected mice are not anoxic [32], thereby preventing the bacilli to be exposed to hypoxic conditions and possibly making anaerobic (nitrate) respiration dispensable in this animal model of tuberculosis. Thus, despite some indirect evidences and correlations, the role of the *narGH* locus in *M. tuberculosis* virulence remains to be demonstrated, and the mechanism(s) involved to be deciphered. Here, we propose that the NarGH-mediated nitrate reductase activity is involved in *M. tuberculosis* virulence and persistence by protecting hypoxic mycobacteria from acid killing, an environmental stress encountered by the pathogen in inflamed granulomatous lesions and cavities [19]. A recent study reported by Sohaskey indicated that exogenous nitrate had no effect on *M. tuberculosis* survival during gradual oxygen depletion (Wayne model), whereas the NarGH-mediated respiratory activity was found involved in *M. tuberculosis* survival during sudden anaerobiosis (and neutral pH), where complete anaerobiosis was achieved within 2 hours and was oxyrase-dependent [33]. In our anaerobiosis model instead, similar to the Wayne model, oxygen depletion is bacterial respiration-dependent and is therefore more gradual since 24 hrs are necessary to achieve complete anaerobiosis, as indicated by methylene blue decoloration. Therefore, the physiology of mycobacteria grown in the sudden anaerobiosis *in vitro* model described by Sohaskey and in ours is likely to be very different, which may not allow direct comparison of the data obtained in both models. In contrast, consistent with Sohaskey's observations in the Wayne model, we found no difference in *M. tuberculosis* survival during anaerobiosis at neutral pH (6.6) with or without exogenous nitrate; the difference was only seen at mild acidic pH (5.5), whereby the presence of exogenous nitrate enhanced *M. tuberculosis* survival.

Nitrate respiration through NarGH activity leads to the production of nitrite which we showed is toxic for the bacterial cell at acidic pH where nitrite is stochiometrically and spontaneously converted into nitric oxide (NO), a potent antimicrobial molecule [28]. To protect itself from nitrite toxicity at acidic pH, we showed that *M. tuberculosis* does not rely on the NirBD nitrite reductase activity for a further reduction of nitrite into ammonium. Instead, nitrite produced upon nitrate respiration is mostly exported outside the cell, likely through predicted nitrite extrusion proteins including NarK3 and NarU [26].

Previous studies have shown that *M. tuberculosis* responds transcriptionally and phenotypically by switching from active division to a nonreplicating state when exposed to NO [8], [14], [17]. NO is a potent antimicrobial molecule that can affect DNA and proteins, including a number of enzymatic activities, in particular haem-containing enzymes, NADH dehydrogenase, succinate dehydrogenase, some metallo-enzymes, and ribonucleotide reductase [34]. Our data show that the NarGH-mediated respiratory activity protects mycobacteria from NO toxicity, thus suggesting that NO does not impair NarGH functionality. Instead, nitrate reductase activity, and hence nitrate respiration, was shown to be upregulated upon NO exposure [35]. We propose that protection against NO stress conferred by the nitrate respiration is mediated by maintenance of the membrane potential and high levels of ATP, the latter being required for nitrosylation-induced repair mechanisms [36]. Alternatively or additionally, complete sequencing of the *M. tuberculosis* genome has revealed the existence of *glb*N and *glb*O genes encoding for distantly related truncated hemoglobins (trHb) N and O, respectively, which catalyze the conversion of NO to nitrate [26]. Previous studies have indeed clearly shown a role of *M. tuberculosis* and *M. bovis* BCG trHbN in NO detoxification [37], [38]. Thus trHb may reveal crucial for the survival of *M. tuberculosis* during infection not only to protect bacteria from NO attack but also to provide the pathogen with an effective TEA for anaerobic respiration.

In conclusion, our work describes a new role for the nitrate respiration and nitrate reductase activity, in protecting hypoxic *M. tuberculosis* against a mild acid challenge. We show that nitrate acts as an efficient TEA that allows hypoxic mycobacteria resist not only to mild acidity but also to NO stress; with the appropriate metabolic enzymes, *M. tuberculosis* appears well-equipped to utilize any available nitrate source for respiration and energy production.
